# Patterns of Impact Resulting from a ‘Sit Less, Move More’ Web-Based Program in Sedentary Office Employees

**DOI:** 10.1371/journal.pone.0122474

**Published:** 2015-04-01

**Authors:** Anna Puig-Ribera, Judit Bort-Roig, Angel M. González-Suárez, Iván Martínez-Lemos, Maria Giné-Garriga, Josep Fortuño, Joan C. Martori, Laura Muñoz-Ortiz, Raimon Milà, Jim McKenna, Nicholas D. Gilson

**Affiliations:** 1 Departament de Ciències de l´Activitat Física, Universitat de Vic-Universitat Central de Catalunya, c/ Sagrada Família 7, 08500, Vic, Barcelona, Spain; 2 Departamento de Educación Física y Deportiva, Universidad del País Vasco, Portal de Lasarte 71, 01007, Vitoria, Spain; 3 Facultad CC.EE. e do Deporte, Universidad de Vigo, Campus A Xunqueira s/n, 36005, Pontevedra, Spain; 4 Physical Activity and Sport Sciences Department, FPCEE Blanquerna, Universitat Ramon Llull, c/Císter 34, 08022, Barcelona, Spain; 5 Physical Therapy Department, FCS Blanquerna, Universitat Ramon Llull. Esport3 Association, c/Padilla 326–332, 08025, Barcelona, Spain; 6 Department of Economics and Business, Universitat de Vic-Universitat Central de Catalunya, c/Sagrada Família 7, 08500, Vic, Barcelona, Spain; 7 Unitat de Suport a la Recerca Metropolitana Nord, Institut Universitari d’Investigació en Atenció Primària Jordi Gol (IDIAP Jordi Gol), c/ Camí del Mig 36, 08303, Mataró, Barcelona, Spain; 8 Departament de Salut i Acció Social, Universitat de Vic-Universitat Central de Catalunya, Vic, Barcelona, Spain; 9 School of Sport, Leeds Beckett University, Fairfax Hall, Headingley Campus, Leeds, LS6 3QS, United Kingdom; 10 The University of Queensland, School of Human Movement Studies, St. Lucia Campus, Brisbane, 4072, Australia; Old Dominion University, UNITED STATES

## Abstract

**Purpose:**

Encouraging office workers to ‘sit less and move more’ encompasses two public health priorities. However, there is little evidence on the effectiveness of workplace interventions for reducing sitting, even less about the longer term effects of such interventions and still less on dual-focused interventions. This study assessed the short and mid-term impacts of a workplace web-based intervention (Walk@WorkSpain, W@WS; 2010-11) on self-reported sitting time, step counts and physical risk factors (waist circumference, BMI, blood pressure) for chronic disease.

**Methods:**

Employees at six Spanish university campuses (n=264; 42±10 years; 171 female) were randomly assigned by worksite and campus to an Intervention (used W@WS; n=129; 87 female) or a Comparison group (maintained normal behavior; n=135; 84 female). This phased, 19-week program aimed to decrease occupational sitting time through increased incidental movement and short walks. A linear mixed model assessed changes in outcome measures between the baseline, ramping (8 weeks), maintenance (11 weeks) and follow-up (two months) phases for Intervention versus Comparison groups.

**Results:**

A significant 2 (group) × 2 (program phases) interaction was found for self-reported occupational sitting (F[3]=7.97, p=0.046), daily step counts (F[3]=15.68, p=0.0013) and waist circumference (F[3]=11.67, p=0.0086). The Intervention group decreased minutes of daily occupational sitting while also increasing step counts from baseline (446±126; 8,862±2,475) through ramping (+425±120; 9,345±2,435), maintenance (+422±123; 9,638±3,131) and follow-up (+414±129; 9,786±3,205). In the Comparison group, compared to baseline (404±106), sitting time remained unchanged through ramping and maintenance, but decreased at follow-up (-388±120), while step counts diminished across all phases. The Intervention group significantly reduced waist circumference by 2.1cms from baseline to follow-up while the Comparison group reduced waist circumference by 1.3cms over the same period.

**Conclusions:**

W@WS is a feasible and effective evidence-based intervention that can be successfully deployed with sedentary employees to elicit sustained changes on “sitting less and moving more”.

## Introduction

Sitting dominates many employees’ work life; 80% of adults in developed countries spend one third of the day in offices doing sedentary, desk-based tasks [1–3]. As a result, over the last 50 years employees´ average daily energy expenditure has decreased by more than 100 kcals [4]. Given the evidence linking prolonged occupational sitting to chronic disease risk and all-cause mortality [5–8], it is clear that developing effective workplace initiatives for reducing total sitting and breaking prolonged bouts of sitting in office workers is an important public health priority [9].

While there is scarce evidence regarding the effectiveness of workplace interventions for reducing sitting [10], there is also a need to explore how evidence-based interventions can be successfully applied in workplaces in order to sustain improvements on employees´ health [11, 12]. Though use of height-adjustable desks and active workstations effectively reduce prolonged occupational sitting time [13–16], and increase standing [13–15] or occupational energy expenditure [16] respectively, that technology involved requires significant changes in the physical office environment and in employees´ work routines. All this can compromise uptake among employers [17]. Most importantly, the lack of evidence regarding the effectiveness of these strategies for sustaining improvements on behavioural risk factors indicates incomplete ‘real world’ understanding of adherence patterns [13–15, 18, 19].

Encouraging workplace walking in office environments may be a practical and feasible means of reducing and breaking occupational sitting time [20]. Notwithstanding that an increase of 2,500 daily steps may reduce daily sitting by 37–45 minutes [21], it remains unclear whether workplace walking initiatives that increase step counts will inevitably reduce occupational sitting over time. Previous studies have assessed the indirect impact of walking strategies on total and occupational sitting time and found no significant intervention effects [10, 20, 22]. While Freak-Poli et al [23] reported reduced sitting time (-0.6 hours/day) among employees who participated in a pedometer-based workplace programme, studies have yet to assess the longer-term impact of workplace sitting and walking interventions on behavioural and physical risk factors for chronic disease [23].

Walk@WorkSpain (W@WS; 2010–11) is an automated ‘sit less, move more’ office-based intervention which targets the prevention and management of chronic disease risk factors. Linked to the international ‘Walk@Work’ initiative, the program provides employees with a pedometer and access to a website which supports them to displace occupational sitting with incidental movement, and short (5–10 minutes) and longer (10+ minute) walks [22]. A recent 10-week evaluation indicated that “Walk@Work” added almost 2,000 extra workday step counts in under-active office workers (<5,000 steps/day) [22].

W@WS builds on these findings and addresses limitations in the current evidence base. Specifically, the present study aims to evaluate program efficacy for the primary outcomes of self-reported sitting, step counts and physical risks factors for chronic disease. Importantly, this study also aims to assess longer-term impacts, within a comparative, rather than pre-post research design in order to show how well an accessible PC-based intervention (W@WS) translates for use in busy office environments.

## Methods

### Study design and sample

The study used a quasi-experimental comparison group pre-post design. Participants were administrative and academic staff working at six campuses in four Spanish Universities in Galicia, the Basque Country and Catalonia (x2). Campuses were randomly assigned by worksite to an Intervention (n = 3; deployed W@WS) or Comparative group (n = 3; maintained normal behavior). In each region, one university campus was randomly assigned to the program (intervention group; IG) and another campus that acted as a comparison group (CG). After assignment, participants were blinded to the existence of other groups receiving different programs. As campuses were located in different cities across Spain, this minimised contamination across groups. The study was approved by the following ethics committee of each university: Ethics Committee of the Faculty in Psychology, Education and Sport Sciences (University Ramon Llull); Research Commission of University of Vic; Ethics Committee of Clinical Research in Conselleria de Sanidad (CEIC; Xunta de Galicia); Ethics Committee of Applied Research in Human Beings (CEISH/GIEB; University of the Basque Country). Participants provided their written informed consent to participate in the study prior to the intervention.

Around 2,500 emails were sent to target campuses. Office workers were first invited to participate in an on-line survey to identify those most in need of intervention (employees located at the low end of the continuum for volume of physical activity) [21, 24]. Physical activity (PA) levels were measured using the International Physical Activity Questionnaire (IPAQ) short form [25]. A total of 704 employees completed the survey [26]. Those employees with low and moderate PA levels (0 to 3,000 MET·min·wk^-1^) were invited to participate in the intervention by email or phone calls (n = 345, 62%). Highly active employees (>3,000 MET·min·wk^-1^) were excluded as they tend to accumulate higher step counts/day [21] and spend lower amounts of time sitting at work [2] than their low or moderately active counterparts. At baseline, both the CG (n = 135) and the IG (n = 129) were given a pedometer and a paper diary to register daily step counts and self-reported sitting time throughout the intervention. During delivery, the IG had access to the W@WS website program while the CG was asked to maintain habitual behavior. The intervention was implemented from September 2010 to June 2011 to fit within the university academic year.

### Intervention

W@WS is an automated web-based intervention that focuses on decreasing occupational sitting time through incidental walking and short walks during the working day. W@WS consists of a ramping (8 weeks) and a maintenance phase (11 weeks). During the ramping phase, every two weeks employees are challenged to progressively increase their movement by 1,000 to 3,000 daily steps above baseline [27, 28]. Strategies to achieve these goals initially focus on breaking occupational sitting time by integrating incidental walking into work tasks (e.g. moving rather than sitting during lectures and seminars, not sitting to take phone calls; weeks 1–2). This progresses to short walks ranging from 5–10 minutes by targeting active mobility within University campuses (e.g. choosing the “longest route” to go to another Department within the campus; weeks 3–4) and then longer walks of +10 minutes by targeting active transport (e.g. walking to work whenever possible) or active lunch breaks (i.e. taking walks after lunch, alone or with colleagues, that fitted within an one-hour lunch break; weeks 5–6). Maps are provided as examples of walks within and around the campus [22, 24, 27]. During weeks 7–8, workers are given information about the extra health benefits of walking faster at a comfortable pace and encouraged to raise their intensity of movement whenever possible (e.g. during active travel or lunch time walks).

During the maintenance period (weeks 9–19), W@WS sends automated emails encouraging workers to sustain sitting reductions and step count increases achieved in the ramping phase. These are sent weekly (weeks 9–12) and then fortnightly; no emails are sent during the last 3 weeks of the program.

W@WS provides a range of ecological support strategies to reduce and break occupational sitting time and increase step counts. These strategies include (a) setting goals every two weeks for increasing step counts as means of reducing occupational sitting time, (b) monitoring the achievement of goals by logging daily step counts into the employee´s personal account (i.e. the resulting graphics provide individual feedback on progress), (c) providing support strategies to achieve the targets and social networking for sharing experiences (i.e. using the blog to share personal strategies for sitting less, walking more and/or ways to overcome personal barriers), (d) increasing employees´ awareness and knowledge of the health benefits of achieving 10,000 steps/day (i.e. preventing weight gain) and reducing sitting time (i.e. providing articles in the web-page published in the mass media or information from well-known scientific organizations), (e) increasing employees´ self-efficacy by suggesting feasible strategies and encouraging them to generate innovative strategies that best enable them to sit less and move more [22, 24, 27].

### Data collection

Trained and experienced researchers implemented a standardized research protocol across the sites. Daily step counts (Pedometer, Yamax-200) and daily self-reported occupational sitting time (paper diary log) were reported during five working days (i) at baseline, (ii) throughout ramping (weeks 1–8) and (iii) maintenance phases (weeks 9–19) and (iv) during two weeks at two months follow-up (week 20–21). Every participant was provided with standard detailed written information on how to use the pedometer and the diary. The physical risk factors measured were body weight and height in light clothing and without shoes (electronic scale—Seca 899/217) and waist circumference (WC) taken at the narrowest part of the torso (directly above the umbilicus) using a flexible steel tape (Seca 203). Systolic blood pressure (SBP) and diastolic blood pressure (DBP) was assessed after the participant sat quietly for 5 minutes (digital automatic blood pressure monitor—Omron M7). At each campus, trained researchers conducted the assessments for the IG and CG at baseline (week 0) and in the final week of each stage using standardized protocols [28]. Demographic details including age, gender and job roles were also recorded during the first scheduled meeting. Trained researchers forwarded SPSS files electronically to a coordinating researcher who pooled and treated the data.

### Statistical analyses

The magnitude of difference (average of weekly measurements on step counts and self-reported occupational sitting) between (i) baseline, (ii) throughout the ramping phase (weeks 1–8), (iii) throughout the maintenance phase (weeks 9–19) and (iv) for two weeks at two months follow-up (weeks 20–21), was used to identify intervention effects across phases on these behavioural risk factors. Employees not providing data at baseline for at least three separate workdays on step counts and self-reported occupation sitting were excluded from the analyses (based on the need to capture the majority of days within a five day working week). A criterion of at least three separate workdays was also applied to the calculation of averages for each phase. Where this criterion was not met, intention to treat was applied and data imputed sequentially using the previously entered average from either baseline or the ramping and maintenance phases as appropriate.

A linear mixed model assessed changes within groups in step counts, self-reported sitting time and behavioural risk factors (WC, BMI, SBP and DBP) across the four program time points. Differences between groups for changes in the main outcomes were assessed using the same model. The model was adjusted by gender and age. The design of the model included participants (fixed factor), group (experimental and comparison group) and program time points (baseline, ramping, maintenance and follow-up). When the interaction between program time points*group was significant, changes 2 x 2 were assessed using post-hoc test adjusted by the Sidak method. Preliminary checks ensured no violation of assumptions of normality, homogeneity of variance and homogeneity of regression slopes.

Binary logistic regression was performed to predict relationships between improved physical risk factors and changes in step counts and self-reported sitting time at maintenance. A first model integrated self-reported sitting time and step count changes into one independent variable adjusted by age and gender (increasing ≥1,000 daily steps and reducing ≥10 minutes sitting a day from baseline). Previous research has reported changes in WC related to 1,000-step incremental changes in step defined PA [29]. Following the criterion of “every minute of sedentary activity is a missed opportunity to accumulate any number of steps taken between 1 and 120 step counts”, replacing 10 minutes of occupational sitting time by 100 step counts/minute could explain an increase in 1,000 daily step counts [30]. A second model contained self-reported sitting time and step count changes as two independent variables adjusted by age and gender. Statistical analyses were performed using PROC MIXED (SAS 9.3 software).

## Results

### Pre-intervention characteristics

**Fig 1:**
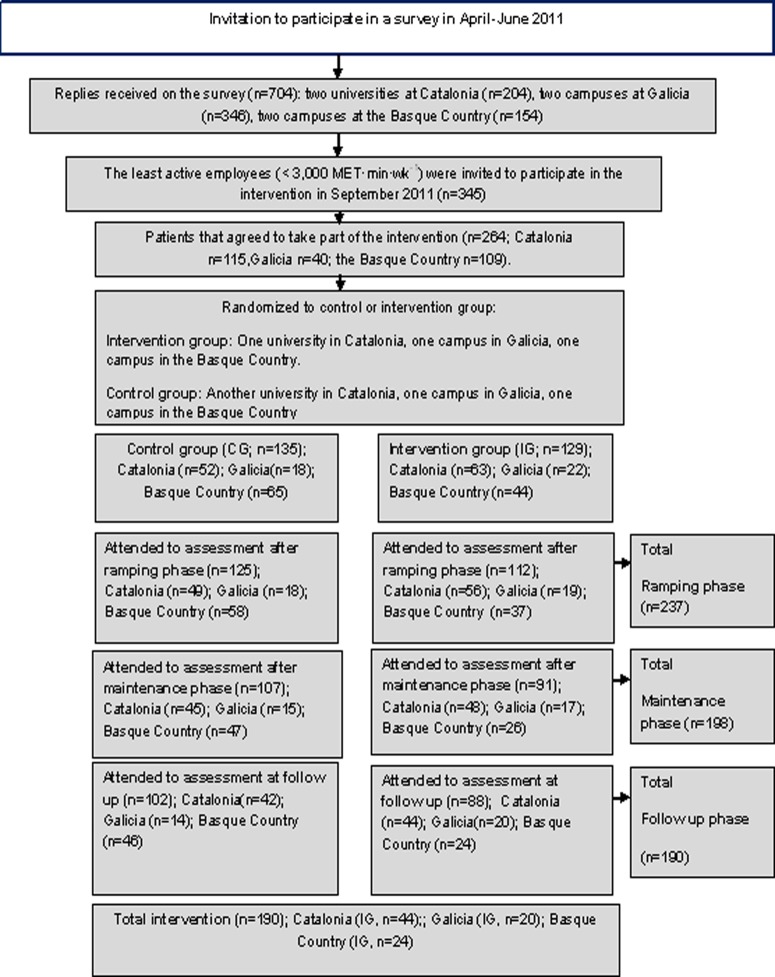
Flowchart of participant´s recruitment across all phases of the intervention.

A total sample size of 264 workers was recruited (42±years of age; n = 171 women; n = 129 administrative staff). In Catalonia, 115 people agreed to participate (IG = 63), with 109 in the Basque Country (IG = 44) and 40 in Galicia (IG = 22). Two hundred and thirty seven employees completed full data measurements from baseline through the ramping period for self-reported occupational sitting time, pedometer-determined step counts and physical risk factors. Full data sets, from baseline through the maintenance period, was provided by 198 (75%) participants, while 190 (72%) completed 19 weeks of data from baseline through follow-up (Fig 1). Drop out after the maintenance period in the Intervention group (n = 38, 29%) was related to sick leave and lack of time (Fig 1).

### Intervention effects on behavioural risk factors

**Fig 2:**
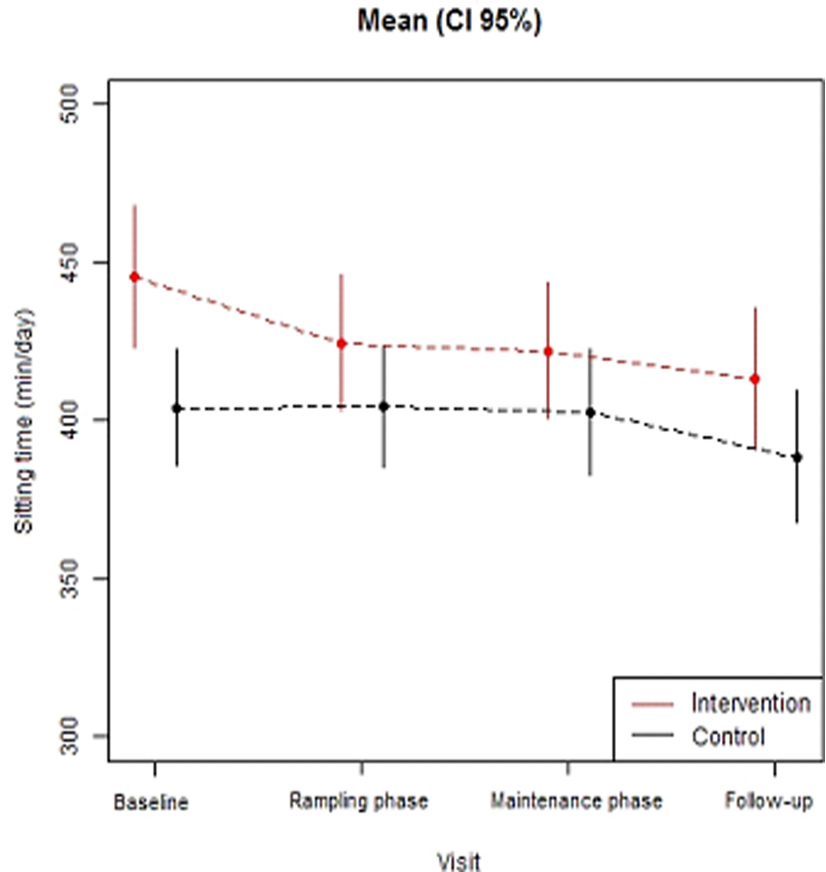
Change in average occupational sitting time for the intervention and comparison groups across program phases.

**Fig 3:**
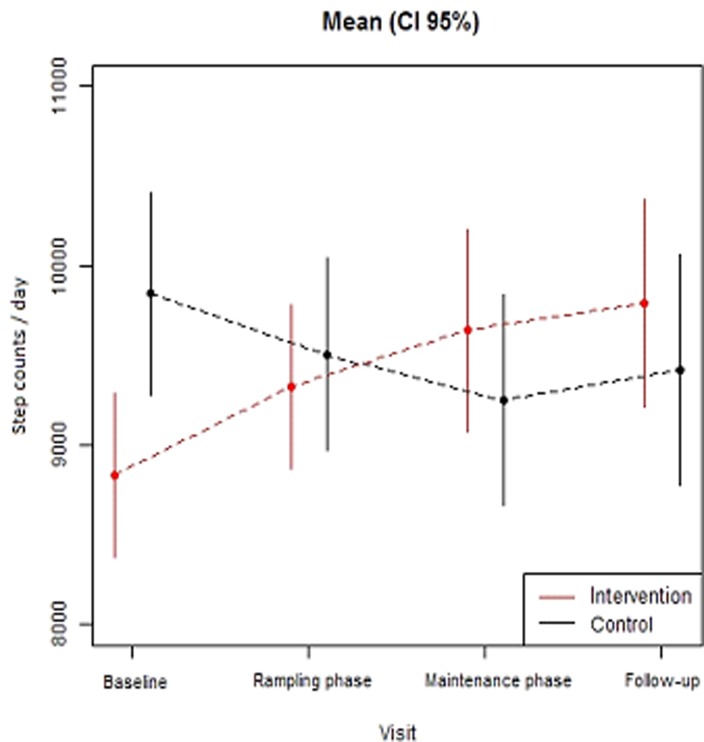
Change in average steps/day for the intervention and comparison groups across program phases.

There was a significant 2 (group) × 2 (program time points) interaction for self-reported occupational sitting (F[3] = 7.97, p-value interaction = 0.046) and daily step counts (F[3] = 15.68, p-value interaction = 0.0013) (Table 1). The IG decreased occupational sitting time from baseline (446.4 minutes ± 126.7) through ramping (425.8 minutes±120.6), maintenance (422.9 minutes ± 123.4) and follow-up (414.2±129.4) (p<0.05) (Table 1), whereas the CG maintained occupational self-reported sitting time through the ramping and maintenance phases but decreased sitting time at follow-up (Table 1; Fig 2). For walking, the IG increased daily step counts across all four program time points (baseline 8,862 ±2,475.75; ramping 9,345±2,435.8; maintenance 9,638±3,131.6 and follow-up 9,786±3,205; Table 1), whereas the CG decreased step counts across the 19 weeks of program delivery and at follow-up compared to baseline (Table 1; Fig 3).

**Table 1:** Outcome measures during the program phases within the Intervention (used W@WS) and Comparison group (maintained habitual behavior).

	Baseline		Rampling phase^a^		Maintenance phase^b^		Follow-up^c^		P values		
	Comparison(n = 135)	Intervention (n = 129)	Comparison (n = 125)	Intervention (n = 112)	Comparison (n = 107)	Intervention (n = 91)	Comparison (n = 102)	Intervention(n = 88)	Group	Program time points	Interaction
Step counts/day; mean (SD)	9920(3484)	8862(2475)	9544(3137)	9345(2435)	9264(3435)	9638(3131)	9427(3744)	9786(3205)	0.372	0.746	0.0013
Sitting time; minutes/day (SD)	404.6(106)	446.4(126)	405.5(110)	425.8(120)	402.8(113)	422.9(123)	388.9(120)	414.2(129)	<0.001	0.048	0.046
Body Mass Index; Kg/m^2^ (SD)	25.9(4.7)	25.5(4.1)	26.1(4.5)	25.6(4.2)	26(4.4)	25.5(4.3)	25.9(4.4)	25.4(4.3)	<0.001	0.711	0.008
Systolic Blood Pressure; mmHg (SD)	122.4(18)	120(16.7)	123.8(17.3)	121.7(16.3)	121.1(16.1)	119.1(15.9)	119(16.5)	117(15.8)	<0.001	0.611	0.995
Diastolic Blood Pressure; mmHg (SD)	78.9(10.8)	77.6(11.9)	78.9(10.6)	78.1(10.5)	77.7(10.3)	77.1(11)	76.7(10.9)	75.7(11.1)	<0.001	0.87	0.898

^a^After the ramping phase (week 8),

^b^After the maintenance phase (week 19),

^c^At two months follow-up (week 21).

Significant differences between groups were found for changes in self-reported occupational sitting time (-22±11 minutes; p<0.005) and workday step counts (+1,432 step/counts; p<0.001; Table 2) at the maintenance phase. At two months follow-up, only increases in step counts remained significant between groups (+1,417 step/counts; p<0.001; Table 2).

**Table 2:** Mean differences relative to baseline across program phases within the Intervention (used W@WS) and Comparison group (maintained habitual behavior) on daily step counts, occupational sitting and waist circumference.

	Intervention Group						Comparison Group					
	Ramping^a^	P-value	Maintenance^b^	P-value	Follow-up^c^	P-value	Ramping^a^	P-value	Maintenance^b^	P-value	Follow-up^c^	P-value
Step counts/day; mean (SD)	+483(145)	0.004**	+776(226)	0.002**	+924(245)	0.000***	-376(174)	0.282	-656(284)	0.202	-493(311)	0.676
Sitting time; minutes/day (SD)	-20.6(6)	0.004**	-23.5(9)	0.040*	-32.2(10)	0.007**	+0.9 (5)	1.000	+1.8(9)	0.999	-15.7(9)	0.414
Waist circumference; cm (SD)	-0.8(0.2)	0.011*	-1.8(0.3)	0.000***	-2.1(0.3)	0.000***	-0.7(0.3)	0.030*	-0.7(0.3)	0.107	-1.30(0.3)	0.000***

^a^After the ramping phase (week 8),

^b^After the maintenance phase (week 19),

^c^At two months follow-up (week 21),

* *p*<0.05,

** *p*<0.01,

*** *p*<0.001.

### Intervention effects on physical risk factors

**Fig 4:**
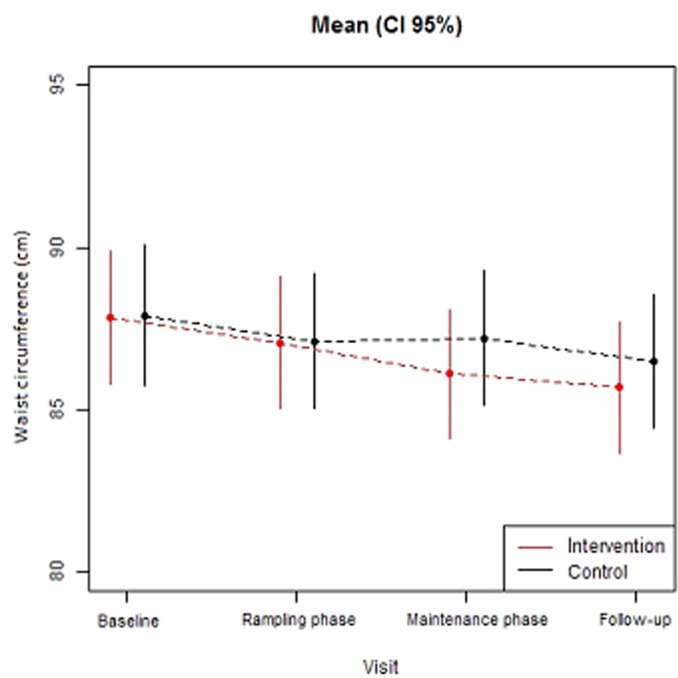
Change in average waist circumference for the intervention and comparison groups across program phases.

There was a significant 2 (group) × 2 (program time points) interaction for waist circumference, F[3] = 11.67, p-value interaction = 0.0086 (Table 1). The IG decreased WC from baseline (85.3 cm ± 13.7) through ramping (84.5 cm±13.8), maintenance (83.5 cm ± 13.7) and follow-up (83.2±13.8) (p = 0.001) (Table 1), whereas the CG reduced waist circumference across program time points with a lower magnitude of change (Table 1; Fig 4). Participants in the IG significantly reduced WC by 1.1cm (p = 0.01) compared to the CG after the maintenance phase and 0.8cms (p = 0.10) at two months follow-up (Table 2). No significant interactions were identified between group and program time points for BMI, SBP and DPB (Table 1).

Employees who combined an increase of ≥1,000 daily steps with a ≥10 minutes/day reduction in sitting time (at maintenance) were twice more likely to reduce WC [OR = 2.07, 95% CI 1.07 to 4.01; *x* ^2^(3, N = 264) 8.08, p = 0.04]. When sitting time and step counts were modelled as independent variables, only step count changes contributed to the model (*x* ^2^[4, N = 264] = 11.82, p = 0.02), yielding an odds ratio of 2.26 (95% CI 1.29 to 3.94). Reductions in waist circumference were not solely influenced by cutting back on self-reported occupational sitting time.

## Discussion

This study assessed the impact of a “sit less, move more” workplace intervention on self-reported occupational sitting time, step counts and physical risk factors over 19 weeks and at two months follow-up. The study provides evidence—which has been called for—that theoretically-derived strategies can be successfully and effectively embedded into workplaces [31]. W@WS represents an effective, low-cost translational program that can be applied to sedentary, desk-based employees. Three main findings stem from identifying the impact of W@WS against a comparison group. First, an automated internet-delivered intervention was effective at reducing occupational daily sitting time while concurrently increasing daily step counts in office employees. Second, beneficial changes in step counts and occupational sitting time were sustained at two months, although only step counts differences between groups remained significant. Third, observed behavioural changes in step counts benefited waist circumference.

The main result of the current study indicated that employees using W@WS decreased daily occupational sitting by 22 minutes/day while also increasing step counts by 1400 steps/day compared to employees in the CG. These findings are similar to previous iterations of W@W established with English-speaking university employees. Although previous studies deployed different program durations and measurement points, occupational sitting time was also reduced (by almost 20 minutes) [20]; and daily step counts increased (averaging +1,400) [22], which gives some indication of the overall potential for change in such employees. Compared to those data, W@WS (i) delivered a longer program (19 weeks vs. 10 weeks), (ii) employed a Comparison group and (iii) was deployed among Spanish university employees. With these features, Walk@Work represents a program with in-built flexibility that can adapt to local environmental and socio-cultural conditions. Data for our Spanish employees reflected that W@WS elicited small and sustainable changes in occupational PA, representing an effective intervention that can be successfully deployed with sedentary Spanish-speaking employees. Eighty-six employees (66.7%) from the IG increased daily step/counts, while 77 (60%) reduced sitting time; for them deploying innovative strategies fitted with daily working routines. This change in routine could help to reduce the health risks related to their sedentary workplace lifestyle.

Recent interventions have reported larger effects on occupational sitting time than W@WS. They showed reductions of 33 minutes sitting per 8-working hours, achieved using height adjustable workstations and 89 minutes and 125 minutes/ 8-working hours day adding an array of other strategies [13, 14]. However, in these interventions workplace sitting was almost exclusively replaced by standing rather than increased step counts, whereas W@WS increased concurrent measures of step counts. Since the energy costs of sitting and standing are similar [17], W@WS represents a potential program for mitigating the diminished energy expenditure inherent to office-based workplaces. Our concurrent data identified that employees from the IG increased energy expenditure by an average of 108 (510; 726) METs–minutes/week when compared to the Comparison group (measured by the IPAQ short-form). Active workstations have been also identified as effective strategies for reducing both occupational sitting time and energy expenditure (~2–4 kcal min^-1^) [16, 17]. However, there are important gaps in the evidence regarding their optimal use and accessibility for both employers´ and workers´ uptake [17]; potentially this compromises longer-term effectiveness in busy office environments. In W@WS, introducing incidental movement and short walks did not seem to interfere with employees´ working routines; the relative small proportion of drop-outs at follow-up from this extended program (n = 41, 32%) seems to confirm this. Therefore W@WS represents a low-cost automated programme, implemented by employees without the need to change the office environment.

W@WS also impacted behavioural risk factors at two months follow-up. While only the increases in step counts remained significant over time between groups, even two months after withdrawing the intervention the IG continued averaging 16.5 minutes less sitting per day at work when compared to the CG. Surprisingly, at two months follow-up our data show that while the percentage of employees increasing daily step counts dropped to 58.9% (-7.8%) the percentage who reduced their sitting time increased to 66.7% (+6.7%). However, since most workplace research has not tracked reductions in total sitting over time, adherence profiles remain unclear [13–15, 18, 19]. Future research should identify the most potent facilitators and barriers influencing individual variability in reducing workplace sitting at the long-term.

Finally, observed changes in step counts and sitting time were significantly associated with an average WC reduction of 1cm after maintenance but not at follow-up. However, WC measurements at follow-up remained lower in the IG compared to the Comparison group (-0.8 cm); with the IG showing a WC reduction of a bigger magnitude than the CG. Our study suggests that improving both behaviors up to specific thresholds (≥1,000 daily steps and ≥10 minutes sitting a day) as well as step counts solely (≥1,000 daily steps) was more likely to predict a WC reduction than reducing sitting time alone over the same threshold. This is consistent with recent cross-sectional evidence identifying no associations between sedentary time and weight outcomes in adults (n = 5,712 adults) [32]. Nonetheless, for a 1cm increase in WC the relative risk of cardiovascular disease events increased by 2% (95% CI = 1–3%) in both men and women [33]; indicating that W@WS can be an effective intervention for chronic disease prevention in sedentary workplaces. Our results also support cross-sectional associations on the added cardio-metabolic health benefits (i.e. reducing waist circumference) of substituting sitting time with MVPA [34, 35].

This study has several strengths and limitations. First, sitting time was measured by self-report. Self-reported sitting time has validity and is an acceptable measure [36], but self-reported measurements might have not been sensitive to detecting all changes in occupational sitting. Future workplace research should use objective measures for sitting time. Nonetheless, W@WS is an original intervention that has evaluated the effectiveness of occupational sitting reduction strategies and increasing walking against a comparison group. This represents the best scientific design for identifying which health interventions achieve the best effects [31]. Secondly, it is important to recognize that this test of W@WS was based on highly educated middle-age men and women working at universities. Ongoing research should focus on more heterogonous samples of office employees from a range of workplaces. However, results from W@WS have identified effective occupational sitting reduction and step counts increase strategies that could be applied to any desk-based occupation. In this regard, W@WS represents a contribution to implementation research that is needed to enhance population health [12].

## Conclusions

W@WS is an evidence-based intervention that successfully encouraged office employees to ‘sit less and move more’, resulting in the improvement of abdominal fatness which is a key physical risk factor for chronic disease. Most importantly, W@WS elicited sustained behavioural changes over time indicating that it is a feasible and effective program for preventing chronic disease in sedentary workplaces. This study contributes to the existing evidence on implementing effective workplace sitting reduction strategies by increasing step counts. The strategies provided by W@WS can be a potential tool to increase office employees´ levels of occupational PA in every day practice. Future research should identify the most potent facilitators and barriers influencing individual variability in reducing workplace sitting at the long-term.
